# Hospitalization and colonization by methicillin-resistant *Staphylococcus aureus* in the surgical department of 03 health facilities in the Ndé division, West-Cameroon

**DOI:** 10.1186/s12941-021-00451-w

**Published:** 2021-07-19

**Authors:** William Lelorel Nguekap Nankam, Pierre René Fotsing Kwetche, Gildas Boris Tazemda-Kuitsouc, Golda Joyce Djeutsa Chouna, Jean Michel Tekam

**Affiliations:** 1 School of Medical Biology, Higher Institute of Health Sciences, Université Des Montagnes, Bangangté, Cameroon; 2 Laboratory of Microbiology, Université Des Montagnes Teaching Hospital, Bangangté, Cameroon; 3 School of Pharmacy, Higher Institute of Health Sciences, Université Des Montagnes, Bangangté, Cameroon; 4 School of Human Medicine, Higher Institute of Health Sciences, Université Des Montagnes, Bangangté, Cameroon; 5 Réseau Des Hygiénistes du Cameroun, Bangangté, Cameroon

**Keywords:** Hospitalization, Methicillin-resistant *Staphylococcus*, Surgical patients

## Abstract

**Background:**

Commensal flora colonization during hospitalization by bacteria is the first step for nosocomial infections while antibiotic resistance reduces therapeutic options. In aim to control this phenomenon, we initiated this study to describe the impact of hospitalization on colonization by methicillin-resistant *Staphylococcus aureus* in the surgical department of 03 health facilities in the Ndé division, West-Cameroon.

**Methods:**

This study was carried out on patients admitted for surgery in 03 health facilities of the Ndé division, West-Cameroon (District Hospital of Bangangté, Protestant Hospital of Bangwa and Cliniques Universitaires des Montagnes). After obtaining ethical clearance and authorizations, nasal swabs were performed at admission and discharge, with the aim of isolating bacteria and performing their antibiotic susceptibility tests. Informations on each participant's antibiotic therapy were recorded. Laboratory investigations were carried out according to standard protocols (CASFM, 2019).

**Results:**

The most commonly used antibiotics were β-lactams. A total of 104 nasal swabs were performed on 52 patients who agreed to participate to the study. From the analysis, 110 (57 at admission versus 53 at discharge) *Staphylococcus* isolates were obtained. Overall, susceptibility testing showed that antibiotic resistance rates were higher at discharge than at admission; with significant differences between the susceptibility profiles obtained at admission and discharge for β-lactams and not significant for fluoroquinolones and aminoglycosides. Globally, frequency of nasal carriage of methicillin-resistant *Staphylococcus aureus* at discharge 16 (30.77%) was significantly higher than at admission 07 (13.46%) with Chi-2 = 4.52 and *p* = 0.0335.

**Conclusion:**

The high rates of antibiotic resistance of bacteria isolated at discharge compared to those isolated at admission obtained in the present investigation, highlights the important role that hospitalization plays in the selection and dissemination of methicillin-resistant *Staphylococcus aureus* and colonization by these bacteria in health structures of Ndé division. As a result, further investigations to find the factors that promote this phenomenon should be carried out.

## Background

*Staphylococcus aureus* are Gram-positive cocci bacteria found on the surfaces of our environment, as well as on the skin and mucous membranes (nasal mucosa) of humans [1, 2]. Mutualist bacteria in humans, they can be pathogenic when the balance is disrupted. Indeed, in presence of a decrease of immunity/injury, these bacteria can cause a multitude of diseases (sepsis, impetigo, endocarditis etc….) more or less severe, while antibiotic resistance reduces therapeutic options. Methicillin resistance was observed in *Staphylococcus aureus* strains in health facilities around 1961 after introduction of this antibiotic, reflecting the role of health facilities in the selection and dissemination of antibiotic-resistant infectious agents [3]. methicillin-resistant *Staphylococccus aureus* isolates can resist to almost all β-lactams [3].

Studies on bacterial resistance in hospitals, more specifically on methicillin-resistant *Staphylococcus aureus* (MRSA) isolates, have highlighted the acquisition of multi-resistant bacteria during hospital stay and the harmful consequences of colonization and infections by these multi-resistant bacteria on patient’s lives [4–6]. Thus justifying the inclusion of the fight against bacterial resistance to antibiotics in global programs (Global Health Security Agenda, One Health) [7, 8]. Also, several studies carried out throughout the world, in West-Cameroon and more precisely in hospitals of Ndé division have shown that the hospital environment (mainly in surgery) is conducive to the selection and dissemination of multi-resistant bacteria on one hand and to the exchange of genetic material between the bacteria in this environment and those in the human body on the other hand [9–13].

Management with antibiotics would become increasingly difficult with emergence of multi-resistant bacteria such as MRSA. Data useful for understanding this phenomenon of growing antibiotic resistance that threatens patient care are scarce around the world and particularly in developing countries where resources (human and financial) for research are limited. However, it’s imperative to understand the evolution of this phenomenon in order to address the morbidity and mortality due to infections by antibiotic-resistant bacteria. It’s in this context and with the aim of making a modest contribution to global programs (Global Health Security Agenda, One Health) focused on the fight against antibiotic resistance that this work was initiated in order to study the impact of hospitalization on MRSA-colonization among patients interned in the surgical department of 03 health care settings in the Ndé division. The results of this investigation will guide patient's antibiotic therapy (Probabilistic antibiotic therapy) at admission and during hospitalization, and will provide a basis for implementing and evaluating strategies and policies to reduce the proportion of antibiotic-resistant bacteria among patients during their hospital stays and thus limit nosocomial infections. This will be done with a view to reduce the emergence of multi-resistant strains in hospitals and their spread from hospitals to the community.

## Materials and methods

### Study site, populations and sampling

This repeated cross-sectional study was conducted from February to May 2019 among patients of surgical departement from admission to discharge in three health facilities of Ndé division, West-Cameroon (District Hospital of Bangangté, Protestant Hospital of Bangwa and Cliniques Universitaires des Montagnes). When all administrative and ethical requirements were met, Clinical data and nasal samples were collected among patients whose consent was given. Clinical data (sex, age, type of surgery, antibiotics administered) were recorded with the help of the attending physicians during hospitalization, while samples were collected at admission and discharge. Nasal swabs were performed by streaking both anterior nares with sterile moistened cotton swabs among patients whose consent was given. Nasal swabs were placed in labelled tubes containing heart-brain broth, stored in an icebox (4–8 °C) and transported without delay to the laboratory of microbiology of Cliniques Universitaires des Montagnes (CUM) for processing.

### Microbiological analysis

#### Bacterial isolation and identification

After seeding specimens on selective culture media (manitol salt agar), we incubated to 37 °C during 18–24 h. Isolation and identification of *Staphylococcus aureus* isolates were based on cultural characteristics, Gram staining, catalase test, coagulase test and DNase test after performing sub-culture on nutrient agar.

#### Antimicrobial susceptibility testing

The susceptibility testing of bacterial isolates against antibiotics was performed by the disk diffusion method (Kirby-Bauer method) on Mueller–Hinton agar according to standard procedures recommended by “Comité d’Antibiogramme de la Société Française de Microbiologie (CASFM 2019)” [14]. After sub-culture on nutrient agar (during 24 h at 37 °C) of a colony isolated on selective media (Manitol Salt Agar), pure culture bacteria obtained has been used to perform a suspension in 0.9% saline with density equal to 0.5 McFarland as recommended by CASFM 2019. The choice of antibiotics was based on those commonly used in the surgical department (after a survey among doctors in the health facilities) and according to the guidelines (CASFM 2019). A total of 10 antibiotics have been used: cefoxitin (30 μg), ciprofloxacin (5 μg), gentamicin (15 μg), norfloxacin (5 μg), penicillin G (10 μg), oxacillin (1 μg), erythromycin (15 μg), clindamycin (2 μg), fusidic acid (10 μg) and co-trimoxazole (1.25/23.75 μg). Interpretations of antibiotic susceptibility results were performed according to the guidelines (CASFM 2019). Reference strains *Staphylococcus aureus* ATCC 29213 were used for quality control.

#### Detection of methicillin-resistant *Staphylococcus* aureus

Phenotypic methods based on susceptibility of oxacillin (1 µg) and cefoxitin (30 µg) had been used for the detection of methicillin-resistant *Staphylococcus aureus* isolates. These were concurrently performed with susceptibility testing of each isolate. Reference strains *S. aureus* ATCC 700699 and ATCC 25923 were used as a positive and negative control, respectively.

### Data analysis

The data collected in current investigation were recorded in Microsoft Excel 2016 software and analyzed with StatView5 software. Descriptive analysis were carried out on the study variables, which included computation of addition and frequencies. The chi-square test was used to compare the susceptibility profiles and frequency of methicillin-resistanct *Staphylococcus aureus* (MRSA) isolates obtained at admission and discharge. The analysis were performed using 95% as confidence interval and 5% as degree of significance.

## Results

### Characteristics of study participants

During this survey carried out from February to May 2019, 52 patients of chirurgical department were recruited. Age of the latter ranged from 5 to 79 years with a mean of 40.77 years. Days of hospitalization were between 3 and 22 with a mean of 7.58. Characteristics of participants were summarized and presented in Table 1.

**Table 1:** Participant’s characteristics

Characteristics	Number	Frequency (%)
Sex		
Men	37	71.15
Women	15	28.85
Type of surgery		
Visceral	38	73.08
Orthopedic	14	26.92
Antibiotics administered		
β-lactams	43	82.69
Ampicillin	21	40.38
Ceftriaxone	29	55.77
Cefixime	9	17.31
Cefuroxime	2	3.85
Amoxicillin/Clavulanic Acid	4	7.69
Cloxacillin	1	1.92
Fluoroquinolones	15	28.85
Ciprofloxacin	15	28.85
Aminosides	17	32.69
Gentamicin	17	32.69
Nitro-5-imidazoles	46	88.46
Metronidazole	46	88.46
Macrolides	1	1.92
Clarithromycin	1	1.92

From Table 1, it appears globally that men represented 3/4 of the participants, visceral surgery 3/4 of chirurgical types, β-lactams and nitro-5-imidazoles were the most antibiotics families administered with proportions approximately equal to 4/5 and macrolides were the least administered with 1/50.

### Distribution of bacterial isolates

From admission to discharge, 104 samples were recorded. Analysis of these samples outcome that 110 *Staphylococcus* isolates were obtained (57 at admission and 53 at discharge). Distribution of *Staphylococcus* isolates is presented in Table 2.

**Table 2:** Distribution of *Bacteria isolates*

Bacteria	Hospitalization			
	Admission		Discharge	
	Number	Frequency (%)	Number	Frequency (%)
*Staphylococcus aureus*	16	28.07	25	47.17
Coagulase negative *Staphylococcus*	41	71,93	28	52.83

Outcome illustrated in Table 2 show a variation between bacteria obtained at admission and those obtained at discharge. Indeed, isolate’s proportion of *Staphylococcus aureus* at discharge 25(47.17%) was greater than admission 16(28.07%).

### Antibiotic susceptibility profiles

The susceptibility profile of bacteria isolated at admission and discharge were huddled in Table 3.

**Table 3:** Antibiotic susceptibility profiles of *bacteria* isolated

	n_admission_ (%)	n_discharge_(%)	*Chi-2*	*p-value*
Cefoxitin (30 μg)				
Susceptible	44(77.19)	14(26.42)	29.49	<0.0001
Intermediate	2(3.51)	2(3.77)		
Resistant	11(19.30)	37(69.81)		
Gentamicin (15 μg)				
Susceptible	52(91.23)	46(86.79)	0.56	0.4559
Intermediate	0(0.00)	0(0.00)		
Resistant	5(8.77)	7(13.21)		
Erythromycin (15 μg)				
Susceptible	18(31.58)	19(35.85)	1.81	0.4051
Intermediate	9(15.79)	4(7.55)		
Resistant	30(52.63)	30(56.60)		
Clindamycin (2 μg)				
Susceptible	21(36.84)	26(49.06)	1.67	0.1957
Intermediate	0(0.00)	0(0.00)		
Resistant	36(63.16)	27(50.94)		
Norfloxacin (5 μg)				
Susceptible	52(91.23)	42(79.25)	3.17	0.0749
Intermediate	0(0.00)	0(0.00)		
Resistant	5(8.77)	11(20.75)		
Ciprofloxacin (5 μg)				
Susceptible	53(92.98)	43(81.13)	3.47	0.0624
Intermediate	0(0.00)	0(0.00)		
Resistant	4(7.02)	10(18.87)		
Fusidic acid (10 μg)				
Susceptible	10(17.54)	4(7.55)	2.47	0.1160
Intermediate	0(0.00)	0(0.00)		
Resistant	47(82.46)	49(92.45)		
Co-trimoxazole (1.25/23.75 μg)				
Susceptible	19(33.33)	17(32.08)	0.02	0.9878
Intermediate	3(5.26)	3(5.66)		
Resistant	35(61.41)	33(62.26)		
Penicillin G (10 μg)				
Susceptible	27(47.37)	3(5.66)	24.09	<0.0001
Intermediate	0(0.00)	0(0.00)		
Resistant	30(52.63)	50(94.34)		
Oxacillin (1 μg)				
Susceptible	43(75.44)	16(30.19)	22.61	<0.0001
Intermediate	0(0.00)	0(0.00)		
Resistant	14(24.56)	37(69.81)		

*n*_*admission*_ number at admission, *n*_*discharge*_ number at discharge

Overall, Table 3 highlights that resistance rates obtained at discharge were higher than those obtained at admission. Significant and insignificant differences between the susceptibility profiles of isolates obtained at admission and discharge were observed.

Significant differences (*p *< 0.0001) were observed for β-lactams: cefoxitin, oxacillin and penicillin G in favour of a higher proportion of resistance to these antibiotics at discharge than at admission, resulting in resistance proportions respectively 4 times, 3 times and 2 times higher at discharge. In contrast, insignificant differences were mainly observed for fluoroquinolones (ciprofloxacin and norfloxacin with *p* = 0.0624, *p* = 0.0749 respectively) and cotrimoxazole (*p* = 0.9878).

### Nasal carriage of methicillin-resistant *Staphylococcus aureus*

A systematic screening of methicillin-resistant *Staphylococcus aureus* was performed during the interpretive reading of the antimicrobial susceptibility testing. Overall 07 methicillin-resistant *Staphylococcus aureus* isolates were obtained at admission versus 16 at discharge. Nasal-carriage distribution of methicillin-resistant *Staphylococcus aureus* by patients are presented in Table 4.

**Table 4:** Nasal carriage of methicillin-resistant *Staphylococcus aureus*

Nasal carriage of MRSA	n_admission_(%)	n_discharge_(%)	*Chi-2*	*p-value*
YES	07(13.46)	16(30.77)	4.52	0.0335
NO	45(86.54)	36(69.23)		

*n*_*admission*_ number at admission, *n*_*sortie*_ number at discharge, *MRSA* methicillin-resistant *Staphylococcus aureus*

From Table 4, it appears that the proportion of nasal carriage of methicillin-resistant *Staphylococcus aureus* at discharge 16(30.77%) was significantly higher than at admission 07(13.46%) with *Chi-2* = 4.52 and *p* = 0.0335.

## Discussion

The aim of this work, conducted between February and May 2019, was to evaluate the impact of hospitalization on colonization by methicillin-resistant *Staphylococcus aureus* among patients interned in the surgical department of a few health facilities in Ndé division.

The majority of patients were male (3/4). This could be justified by the fact that they are the most regularly involved in hazardous activities, requiring great physical effort and therefore more frequently victims of accidents of all kinds. These activities expose them to accidents that can only be remedied by invasive acts such as surgery. This view is shared by the proportion of visceral interventions for hernias. Other causes that are not clear from this work but which are most probably at the origin of internalizations in surgical departments include road accidents and those associated with other high-risk professions.

Data analysis indicated that more than 80% of the most commonly administered antibiotics to patients were nitro-5-imidazoles and β-lactams. Several reasons could explain this rate of use: 1. these antibiotics have a wide spectrum of action on several bacterial types, have good tissue diffusion and are the most available and accessible in hospital pharmacies; 2. the less frequent adverse reactions (toxicity) to their administration encourage their choice in the management of patients during prophylaxis and anti-infectious therapy [15, 16]. This development is further supported in the present work by the rates of use of other antibiotics (macrolide), which are certainly lower in relation to their relatively limited spectrum of Gram-positive bacteria and high toxicity [15].

Different frequencies were recorded for *Staphylococcus aureus* and coagulase-negative *Staphylococcus* at admission and discharge. 16 (28.07%) *Staphylococcus aureus* isolates were obtained at admission versus 25 (47.17%) at discharge. This result could be justified, at least in part, by a deselection of coagulase-negative *Staphylococcus* in favour of *Staphylococcus aureus*. But the mechanisms and conditions conducive to this selection are far from being clarified in the present work.

From the study of susceptibility profiles of isolates to the antibiotics used and more specifically to methicillin (highlighted in the present work using oxacillin and cefoxitin), it appears that resistance rates recorded at discharge were higher than those observed at admission. Indeed, proportion of nasal carriage of methicillin-resistant *Staphylococcus aureus* at discharge 16 (30.77%) was significantly higher than at admission 07(13.46%) with *p* = 0.0335. This result could be justified by the mobility of genetic factors, favoured both by the selection pressure imposed by broad spectrum antibacterial agents and the flexibility of the bacterial genome. These same factors could explain the multiple resistances regularly reported [9, 10] in accordance with the results of this study [9, 10, 12, 17–20].

Between admission and discharge, significant differences (*p* < 0.0001) concerning susceptibility profiles had been observed for antibiotics belonging to the β-lactams family (cefoxitin, oxacillin and penicillin G). This significant difference was in favour of a 4 times, 3 times and 2 times respectively higher resistance at discharge. This result could be justified by the direct effect of the use of antibiotics belonging to the β-lactams family (most commonly used antibiotics) in the selection and expression of resistance genes such as the *mec A* gene, which codes for methicillin resistance and induces resistance to practically all antibiotics in the β-lactams family. [1, 20–23].

These resistances are an alert as to the difficulty that would exist in managing a resistant infection and the need to use an antibiotic susceptibility test for therapeutic choice (personalization of management) with the antibiotics available and accessible in the target hospital settings in this work.

This result could be justified at least in part by a flaw in the respect of hygiene rules and the importance of the selection and dissemination of multi-resistant bacteria in the hospital environment.

Emergence and dissemination of bacteria resistant to antibiotics in hospitals as demonstrated in this investigation is an indirect indicator of the increasing additional cost to the patient. Given the standard of living and purchasing power, this evolution of resistance would be seen as a factor aggravating poverty through prolonged hospital stay and the cost of care. Taking into account the susceptibility profile at the patient's entry could be a major asset for drug management using antibiotics.

## Conclusion

At the end of our investigation, the aim was to evaluate the impact of hospitalization on colonization by methicillin-resistant *Staphylococcus aureus* among patients interned in the surgical department of 03 care settings in the Ndé division. From this, it appears that the rates of antibiotic resistance of *Staphylococcus* isolates obtained at discharge were globally higher (mainly with regard to antibiotics belonging to the β-lactams family, the most used family) than those obtained at admission and that colonization by methicillin-resistant *Staphylococcus* was more important at discharge than at admission. Thus, this work must be considered as an alarm bell with regard to the emergence and spread of resistant bacteria in Ndé division in hospital and hospitals to the community. As a result, further investigations to find the factors that promote this phenomenon should be carried out.

## Data Availability

All data generated or analyzed during this study are included in this published article.

## Abbreviations

ATCC: American Type Culture Collection
CASFM: Comité de l’Antibiogramme de la Société Française de Microbiologie
*Chi-2*: Chi-square test
MRSA: Meticillin-resistant *Staphylococcus aureus*
